# Automated Morphological Profiling via Deep Learning-Based Segmentation for High-Throughput Phenotypic Screening

**DOI:** 10.3390/jimaging12040179

**Published:** 2026-04-21

**Authors:** Bendegúz H. Zováthi, Philipp Kainz

**Affiliations:** 1 Faculty of Information Technology and Bionics, Pázmány Péter Catholic University, 1083 Budapest, Hungary; 2 KOLAIDO GmbH, Flughafenstrasse 11, 9423 Thal, Switzerland

**Keywords:** morphological profiling, Cell Painting, high-content screening, deep learning segmentation, instance segmentation, microscopic image analysis, phenotypic screening, high-throughput imaging, artificial intelligence, drug discovery

## Abstract

Reproducible morphological profiling, particularly for drug discovery, has become an important tool for compound evaluation. Established workflows such as CellProfiler provide a widely adopted foundation for Cell Painting analysis. However, conventional pipelines often require substantial manual configuration and technical expertise, which can limit scalability and accessibility. In this study, a fully automated deep learning-based workflow is presented for segmentation-driven morphological profiling from raw microscopy data. Using a curated subset of the JUMP Cell Painting pilot dataset, ground-truth masks were generated and used to train a U-net–based segmentation model in the IKOSA platform. Post-processing strategies were introduced to improve instance separation and reduce segmentation artifacts. The final model achieved strong segmentation performance (precision/recall/AP up to 0.98/0.94/0.92 for nuclei), with an average runtime of 2.2 s per 1080 × 1080 image. Segmentation outputs enabled large-scale feature extraction, yielding 3664 morphological descriptors that showed high correlation with CellProfiler-derived measurements (normalized MAE: 0.0298). Feature prioritization further reduced redundancy to 1145 informative descriptors. These results demonstrate that automated deep learning pipelines can complement established Cell Painting workflows by reducing configuration overhead while maintaining compatibility with validated morphological profiling standards. The proposed workflow may help improve resource efficiency in drug discovery and personalized medicine.

## 1. Introduction

### 1.1. Problem Statement

Morphological profiling has become an important tool in drug discovery and precision medicine, enabling systematic evaluation of compound effects at the cellular level [1,2]. Advances in high-throughput screening technologies have dramatically increased the volume and complexity of microscopy data, creating a growing demand for automated and scalable analysis pipelines [3]. Modern imaging platforms allow researchers to acquire large quantities of high-resolution cellular data; however, the analysis of these datasets remains a major bottleneck due to the complexity of segmentation and feature extraction workflows.

Among high-content imaging techniques, the Cell Painting assay [1] has emerged as a widely adopted protocol for capturing rich morphological features of cellular perturbations. Developed at the Broad Institute of MIT and Harvard [4] by the Carpenter [5] and Schreiber laboratories [6], Cell Painting combines multiplexed fluorescent staining with image analysis to characterize cellular phenotypes. The Joint Undertaking in Morphological Profiling (JUMP-CP) consortium [7] has further advanced this field by generating large public reference datasets including millions of images and perturbations [3]. These datasets enable systematic investigation of compound activity, mechanism-of-action prediction, and phenotype-based drug discovery.

The primary computational backbone of the Cell Painting [1] workflows is CellProfiler [8,9], an open-source image analysis platform that provides tools for image correction, segmentation and large-scale feature extraction. By transforming microscopy images into high-dimensional quantitative descriptors, CellProfiler [8,9] enables researchers to address biological questions in a data-driven manner. Despite its widespread adoption, the analysis workflow often requires careful pipeline configuration, parameter tuning, and domain expertise, which may limit accessibility for non-expert users.

The increasing amount of high-content microscopy data raises the need for automated and robust analysis pipelines that minimize manual intervention while maintaining accuracy and reproducibility. Segmentation is a particularly critical step because errors introduced at this stage directly influence the extracted morphological features and their biological interpretation. Therefore, the motivation of this work is to develop a fully automated computer vision–based workflow for Cell Painting [1] analysis that reduces configuration complexity and makes morphological profiling more accessible to researchers without a computer science background.

### 1.2. Related Work

Image segmentation is a fundamental task in computer vision and biomedical image analysis, aiming to assign object labels to individual pixels. Deep learning architectures such as fully convolutional networks (FCNs) [10] and U-net models [11] have become standard approaches for biomedical segmentation tasks due to their ability to capture contextual and spatial information.

More advanced frameworks, including region-based detectors such as Faster R-CNN [12] and Mask R-CNN [13], enable instance-level segmentation by combining object detection and pixel-wise classification. Alternative real-time detection models such as YOLO [14] have further accelerated progress in object detection and segmentation research. Recent developments also include Transformer-based and hybrid architectures that improve segmentation performance in complex visual scenes [15,16,17,18]. Foundation models such as the Segment Anything Model (SAM) [19] further demonstrate the potential of training robust segmentation models across domains.

In the domain of microscopy, specialized models such as CellPose [20] have demonstrated strong generalization across imaging modalities by leveraging large and diverse training datasets. Despite these advances, many general-purpose cell segmentation approaches are not directly optimized for morphological profiling workflows such as Cell Painting [1], where accurate compartment-level segmentation and compatibility with feature extraction pipelines are essential. This motivates the development of domain-specific, automated solutions that integrate segmentation with downstream morphological analysis.

## 2. Materials and Methods

### 2.1. Dataset Description

The primary data source for this study was the Cell Painting Gallery (CPG) dataset [21], which was generated using CellProfiler [9]. The dataset contains fluorescence microscopy images (TIFF), segmentation outlines (PNG), extracted morphological features (CSV), and metadata (CSV/TXT). It is maintained by the Carpenter–Singh Lab [5] and the Cimini Lab [22] at the Broad Institute [4]. The data are publicly available under the CC0 1.0 license via the AWS Registry of Open Data [21].

The Joint Undertaking in Morphological Profiling—Cell Painting (JUMP-CP) [7] released multiple datasets, including the large-scale CPG0016 reference dataset and several pilot datasets designed to evaluate perturbation conditions [23]. For this study, the CPG0000-jump-pilot dataset [24] was used, which contains chemical and genetic perturbations across multiple experimental settings [3]. The dataset includes Cell Painting data generated from A549 lung carcinoma epithelial cells and U2OS osteosarcoma-derived epithelial cells.

#### 2.1.1. Experimental Parameters

The dataset includes 51 plates acquired in a 384-well format (16 × 24 layout), with 2–4 biological replicates and time points of 24, 48, 96, and 144 h. Treatments include chemical compounds, Clustered Regularly Interspaced Short Palindromic Repeats single-guide RNAs (CRISPR sgRNAs), and Open Reading Frame (ORF)-based perturbations targeting more than 175 genes. In total, 5794 extracted features describe cellular morphology across multiple compartments (Table 1).

#### 2.1.2. Image Organization

The dataset is hosted on AWS S3 [21]. Image files are organized by acquisition center and plate, with standardized naming encoding row, column, field of view, and channel. Images are 16-bit, 1080 × 1080 pixel TIFF files. Illumination correction files and CellProfiler [9] workspaces are also provided.

**Figure 1:**
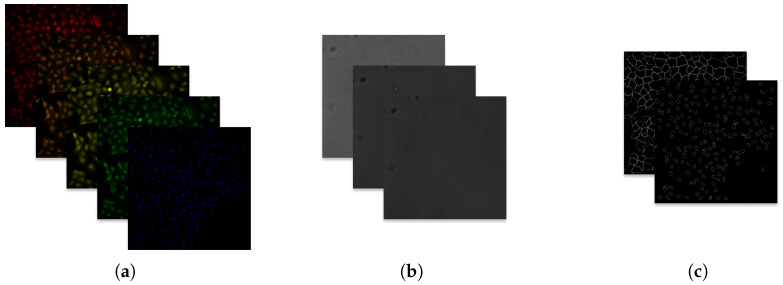
Example Cell Painting image data (BR00116991-A02-5). (**a**) Fluorescent channels. (**b**) Brightfield channels. (**c**) Segmentation outlines.

Five fluorescent dyes label key cellular compartments (Figure 1a, Table 2), complemented by three brightfield channels capturing texture (Figure 1b). Segmentation annotations and tables of morphological features generated by CellProfiler [9] are available as outline images and CSV files (Figure 1c).

#### 2.1.3. Morphological Feature Extraction

Morphological features were generated using CellProfiler [9]. Three compartments were analyzed: the nucleus, cytoplasm and whole cell. Segmentation is based on the DNA channel for nuclei, RNA for cells, and derived by subtraction for the cytoplasm. Features are grouped into seven categories: AreaShape, Correlation, Granularity, Intensity, Neighbors, RadialDistribution and Texture [25]. According to Cimini et al. [26], 1774 features are typically extracted per image, with distributions shown in Table 3.

#### 2.1.4. Subset Selection

**Figure 2:**
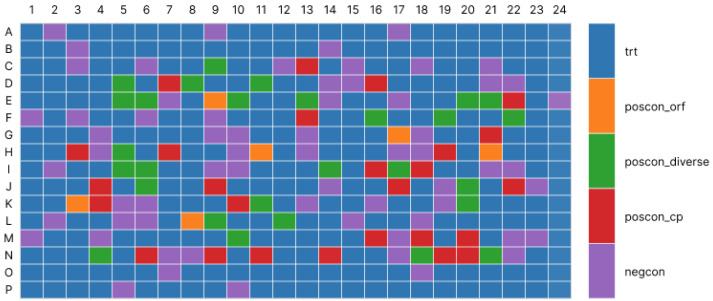
Plate map of compound plates (blue: treatments; orange, green and red: positive controls; purple: negative controls).

To ensure robust model development, careful selection of the training dataset was essential, as data quality directly determines segmentation performance. To maximize phenotypic diversity, a representative subset of compound-treated plates was selected (Figure 2).

In this subset, A549 and U2OS cell lines were captured at 24 and 48 h under multiple perturbation settings. Each plate contains 384 wells (16 rows and 24 columns) representing hits, diverse non-hits, and positive and negative controls. Hits were defined as wells significantly deviating from the negative control median, while controls ensured assay validity and experimental consistency. From each plate, the same well positions were selected to increase the variations of phenotypes, containing 28 hits, 19 non-hits but diverse phenotypes, 10 positive controls and 64 negative controls, corresponding to 121 wells overall.

Wells were typically captured in nine fields of view. For consistency and to avoid border artifacts, the central field of view was selected. The final dataset consists of 2783 multi-channel images with corresponding CellProfiler [9] outputs (90 GB). Data preprocessing and preparation for segmentation model training are described in Section 2.2.2.

### 2.2. Overview of the Workflow

This study develops an automated Cell Painting [1] image analysis workflow combining deep learning-based segmentation and segmentation-driven feature extraction. Starting from multi-channel microscopy images and CellProfiler-generated annotations [9], a deep neural network was trained to predict segmentation masks for nuclei and cells. Based on the predicted instance masks, morphological features were extracted to describe cell compartments. The end-to-end pipeline was validated by comparing segmentation performance and downstream feature measurements against the state-of-the-art Cell Painting [1] protocol implemented with CellProfiler [9]. The workflow consists of: (i) generating ground-truth labels from the JUMP-CP [7] pilot dataset [24] (Section 2.2.2), (ii) training a deep learning segmentation model on a curated subset (Section 2.3), and (iii) extracting morphological features and benchmarking them against CellProfiler [9] (Section 2.4).

#### 2.2.1. Metrics

Segmentation quality was evaluated using the Intersection over Union (IoU), defined as the overlap between a prediction and ground truth (Equation (1)), where 0 indicates no overlap and 1 indicates perfect agreement:

(1) $$\Phi \left(\Lambda^{G},\Lambda^{P}\right)={\left\{\phi_{t}\right\}}_{t=1}^{N},\phi_{t}=\frac{R_{t}^{G}\cap R_{t}^{P}}{R_{t}^{G}\cup R_{t}^{P}}$$

where $\Phi \left(\Lambda^{G},\Lambda^{P}\right)={\left\{\phi_{i}\right\}}_{i=1}^{N}$ denotes the set of per-element similarity scores between ground truth ($\Lambda^{G}$) and predictions ($\Lambda^{P}$), where each $\phi_{i}=\frac{R_{i}^{G}\cap R_{i}^{P}}{R_{i}^{G}\cup R_{i}^{P}}$ is the intersection over union between the *i*-th ground-truth region ($R_{i}^{G}$) and the predicted region ($R_{i}^{P}$).

Object-level performance was summarized via a confusion-matrix formulation (TP/TN/FP/FN), enabling determination of the Average Precision (AP), Precision (P), Recall (R), and F1 score (F1) [27]. True positives were defined as valid object matches when the IOU exceeded a chosen threshold. Average precision was computed as follows:

(2) $$AP=\frac{TP}{TP+FP+FN}$$

Recall and precision were computed as follows:

(3) $$R=\frac{TP}{TP+FN}$$

(4) $$P=\frac{TP}{TP+FP}$$

The F1 score was computed as follows:

(5) $$F1=2\times \frac{P\cdot R}{P+R}$$

To quantify differences between measured and reference feature values, Mean Squared Error (MSE) and Mean Absolute Error (MAE) were used:

(6) $$MSE\left(y,\hat{y}\right)=\frac{1}{n}\sum_{i=1}^{n}{\left(y_{i}-\hat{y}i\right)}^{2}$$

(7) $$MAE\left(y,\hat{y}\right)=\frac{1}{n}\sum_{i=1}^{n}\left|y_{i}-{\hat{y}}_{i}\right|$$

#### 2.2.2. Preprocessing the Data

A representative subset of the JUMP-CP [7] pilot dataset [24] was selected to maximize phenotype diversity. The subset consisted of the central field of view from 121 wells across 23 compound plates (2783 multi-channel image samples in total), corresponding CellProfiler [9] outline annotations, metadata and feature outputs. All of this data was downloaded from the Cell Painting Gallery AWS S3 Bucket [21].

**Figure 3:**
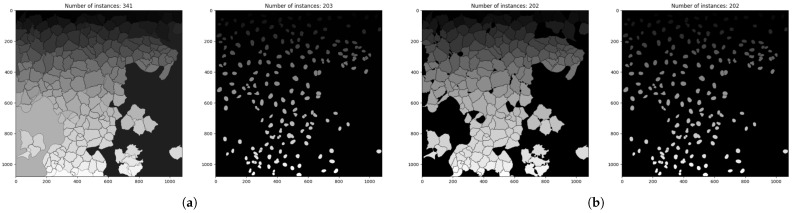
Executing the preprocessing methods on image BR00116991-A01-1. (**a**) Cell and nucleus masks after inversion and connected component analysis. (**b**) Cell and nucleus masks after filtering out background and redundant objects. Masks use instance-label encoding, where each connected component is assigned a unique grayscale value and the numbers above indicate the total instance count.

Cell Painting Gallery [21] annotations are provided as segmentation outlines, requiring preprocessing for supervised segmentation training. Each outline is a single-channel PNG with border pixels at a value of 255 and the background at 0. Ground-truth instance masks were generated using the following procedure:

1. Outline inversion to obtain filled masks;
2. Connected component analysis to identify individual instances (Figure 3a);
3. Removal of redundant objects based on CellProfiler [9] segmentation outputs (Figure 3b);
4. Morphological dilation applied to restore border pixels (kernel size was tuned based on the mean object area provided by CellProfiler [9]).

**Figure 4:**
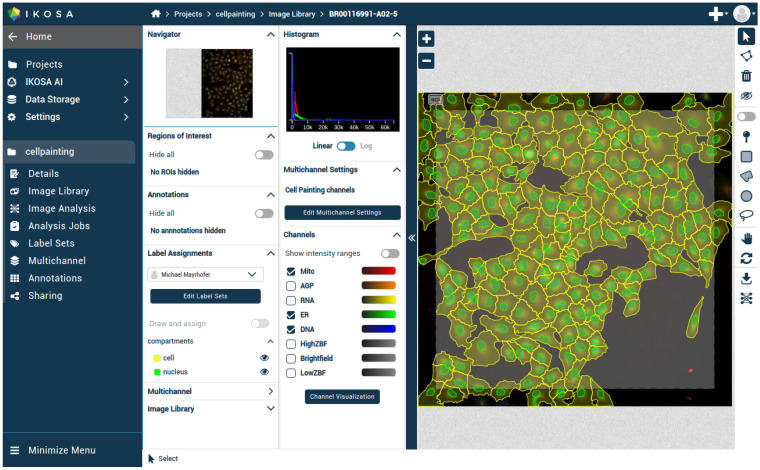
BR00116991-A02-5 image and annotations on IKOSA platform [28].

The preprocessed data was uploaded to the IKOSA [28] platform using a custom multithread script that fused channels and uploaded ground-truth labels as polygons. A total of 568,083 annotation polygons were uploaded. To avoid incomplete objects at image borders (e.g., cells missing nuclei), a 960 × 960 pixel region of interest (ROI) was defined at the center of each 1080 × 1080 image. After polygon upload, IKOSA [28] generated masks from polygons (Figure 4). Image names encoded plate, well, and field-of-view identifiers (e.g., BR00116991-A02-5). The dataset was split into non-overlapping training (*n* = 2208 images) and validation (*n* = 572 images) sets by randomly selecting approximately 80/20% of wells from each plate.

### 2.3. Training IKOSA AI Instance Segmentation Model

A supervised learning approach was used to predict pixel-wise segmentation outputs from the uploaded multichannel images and ground-truth instance annotations.

**Figure 5:**
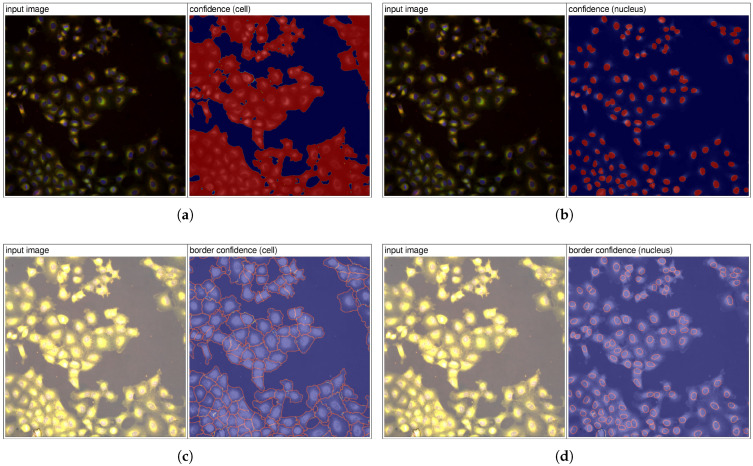
Confidence maps for image BR00116992-N14-5. Panels (**a**,**b**) show class confidence maps, where each pixel encodes the probability of belonging to a cell (**a**) or nucleus (**b**), with warmer colors indicating higher confidence and cooler colors representing the background. Panels (**c**,**d**) present border confidence maps, where pixel intensities reflect the likelihood of being on object boundaries, highlighting cell (**c**) and nucleus (**d**) contours and instance separation.

AI instance segmentation training was performed through the IKOSA AI GUI [28] with the following configuration: segmentation type—“instance”; labels—“cell”, “nucleus”; train/validation split—“random”; image type—“multichannel”; training duration—“extended”. For accurate instance separation, the model predicts confidence maps for instance areas and corresponding borders (Figure 5). During inference, these confidence maps are post-processed using heuristics based on the Watershed algorithm [29].

Empirical evaluation indicated that semantic segmentation for cells, combined with instance segmentation for nuclei, provided robust initial outputs. To address split-and-merge errors in cell instances, two post-processing procedures were applied:

1. Splitting multi-nuclear cell instances: Cell instances containing more than one nucleus centroid were detected (assuming predominantly mononuclear cells). Cell regions were partitioned by assigning pixels to the nearest nucleus centroid using Euclidean distance-based nearest-neighbor assignment.
2. Merging fragmented cell instances and noise removal: The second algorithm evaluates detected cells that contain no nucleus and are below a certain area. Poorly connected regions were classified as noise and were removed. Fragments adjacent to other cells were merged with the most probable neighboring cell based on border-confidence information.

### 2.4. Feature Extraction and Quality Control

**Figure 6:**
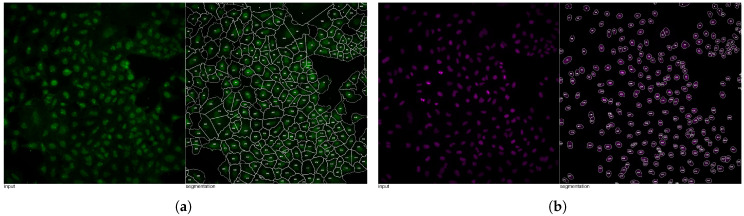
Object masks used as input for feature extraction for image BR00116991-A09-5. (**a**) Cell segmentation outputs. (**b**) Nucleus segmentation outputs.

Morphological features were extracted from the instance segmentation output (using predicted cell and nucleus masks as inputs (Figure 6)) to the final model (CEL-39). Features were grouped into: area and shape (including Zernike features), correlation, granularity, intensity, location, neighbors, radial distribution, and texture (Haralick).

To benchmark feature extraction, CellProfiler [9] was used with the JUMP analysis v3 pipeline [30,31], which was extended to cover the feature set of the JUMP-CP [7] pilot dataset [24]. Running the pipeline produced reference outputs for cell and nucleus objects.

Using these extracted feature groups, dimension reduction was performed based on correlation across cell and nucleus outputs. Among 3664 total features, 31% were prioritized, resulting in a subset of 1145 descriptive parameters for phenotype profiling and hit selection using statistical correlation to remove redundancy.

## 3. Results

This section evaluates the performance of the proposed segmentation pipeline using both quantitative metrics and qualitative assessment. Multiple training configurations were explored to optimize nucleus and cell instance segmentation.

The initial model (CEL-36) was trained using the default IKOSA [28] extended training configuration with early stopping (training time: 4 h 22 min). The model achieved accurate semantic segmentation, with IoU values of 0.89 for nuclei and 0.62 for cells, but showed limited instance separation capability. While nucleus instance segmentation achieved an Average Precision (AP) of 0.89, cell instance segmentation remained low (AP = 0.44), particularly in dense cellular regions where objects were frequently merged.

To improve segmentation quality, the training configuration was extended by increasing the maximum training duration (up to 120 h), adjusting epoch steps (to 500) and early stopping patience (to 1000 epochs), and slowing learning-rate decay. These modifications enabled more stable convergence and reduced the likelihood of stopping at local minima. The resulting model (CEL-37) achieved improved nucleus segmentation (AP = 0.93) and a higher cell IoU (0.72) but still exhibited two dominant failure modes: merged cell instances and false-positive detections in crowded regions.

**Figure 7:**
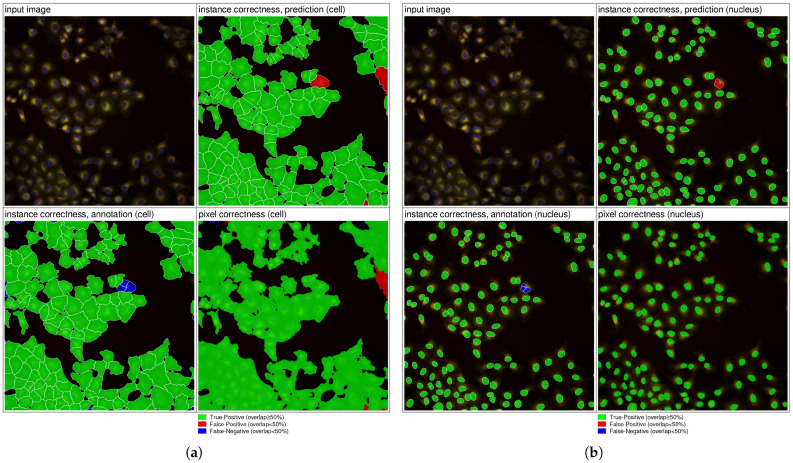
Segmentation results for BR00116992-N14-5. (**a**) Cell segmentation. (**b**) Nucleus segmentation.

To address these issues, advanced post-processing algorithms were introduced to align cell and nucleus predictions. These included the splitting of multi-nuclear cell regions and merging of small false-positive fragments based on border-confidence maps. The qualitative impact of these refinements is illustrated in Figure 7, demonstrating improved instance separation and reduced false-positive detections. The refined model (CEL-39) substantially reduced false-positive cell detections (Figure 7a) while maintaining strong nucleus segmentation performance (Figure 7b). Instance correctness is determined based on IoU overlap with the ground truth: predictions with IoU ≥0.5 are counted as true positives, predictions with IoU <0.5 are considered false positives, and ground-truth objects without a matching prediction at IoU ≥0.5 are counted as false negatives. Pixel correctness reflects the per-pixel correspondence with the annotation.

**Figure 8:**
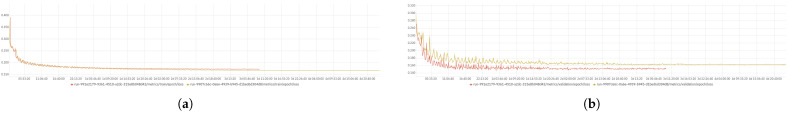
Training and validation loss using 8 and 5 channels. (**a**) Training loss for CEL-39 (red) and CEL-56 (yellow). (**b**) Validation loss for CEL-39 (red) and CEL-56 (yellow).

To evaluate the influence of input channels, an additional model (CEL-56) was trained using only the five fluorescent channels. Segmentation performance remained comparable to earlier baseline models, indicating that brightfield channels provide limited additional benefit for instance segmentation. This suggests that the relevant structural information required for segmentation is already sufficiently captured by the fluorescence channels. Training and validation loss curves comparing the eight-channel and five-channel models are shown in Figure 8.

The final instance segmentation model (CEL-39) was trained using eight-channel inputs on a curated subset of the JUMP-CP [7] pilot dataset [24]. The training set included 2208 images containing 215,732 nucleus and 231,501 cell annotations, while the validation set contained 572 images with 58,290 nuclei and 62,560 cell labels. The model processed full-resolution images (1080 × 1080 pixels) in approximately 2.2 s per image on a single GeForce GTX 1080 Ti GPU.

Quantitative evaluation showed strong performance on the ground truth, as summarized in Table 4. The model achieved high overlap for both nuclei and cells, with high precision and recall values and relatively low false-positive counts, demonstrating robust instance-level segmentation across plates and experimental conditions.

**Figure 9:**
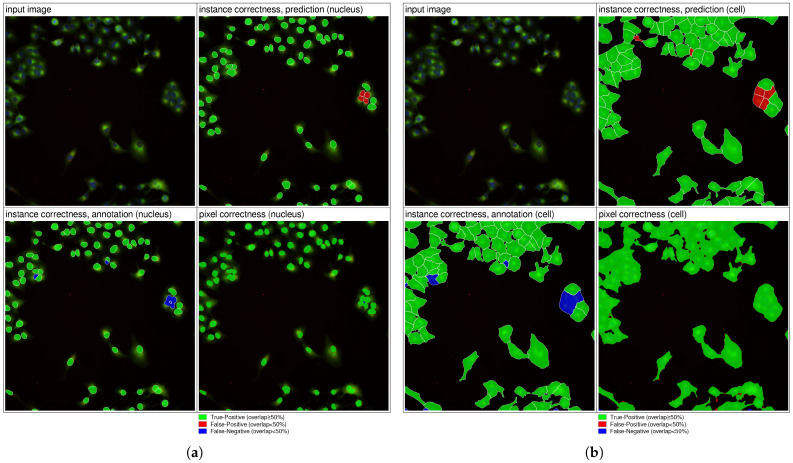
Segmentation results for BR00117008-C03-5. (**a**) Nucleus segmentation. (**b**) Cell segmentation.

Qualitative evaluation confirmed accurate nucleus segmentation, even in challenging cases (Figure 9a). In some instances, predictions appeared more consistent than the provided annotations, suggesting minor inaccuracies in the reference labels. Cell segmentation benefited from nucleus-guided instance separation (Figure 9b), although a small number of false negatives remained. Overall, qualitative observations suggest that the effective segmentation performance may be slightly underestimated by quantitative metrics, potentially reflecting limitations of the ground-truth annotations.

## 4. Discussion

Morphological profiling has become an important part of drug discovery and precision medicine, enabling large-scale characterization of cellular responses based on measurable phenotypic changes. The Cell Painting assay [1] was designed to provide a framework for morphological profiling, with the long-term goal of enabling non-programming users to analyze high-content imaging data with minimal configuration effort. Despite these advances, practical deployment remains challenging, particularly due to the complexity of segmentation workflows and the dependency on parameter tuning.

CellProfiler [9] is the current state-of-the-art solution for Cell Painting-based analysis [1] due to its extensive feature extraction capabilities and flexibility. However, several limitations persist. Processing large datasets requires substantial computational resources, and segmentation accuracy strongly depends on image quality and parameter configuration. Developing optimized pipelines often requires scripting expertise and iterative fine-tuning. Moreover, the reproducibility of workflows may be limited by the dependency on user-defined settings, making standardization across experiments difficult. As discussed by the Carpenter–Singh Lab [32], determining whether a pipeline is “good enough” depends on application-specific trade-offs between accuracy, time, and robustness. In practice, researchers often adapt existing pipelines and iteratively refine parameters using test datasets before scaling to full experiments, which requires time-consuming manual optimization.

The broader field has also highlighted the significant configuration effort associated with segmentation methods. As illustrated in Table 5, configuring segmentation workflows can require substantial time investment depending on the method and user expertise. Even tools designed to reduce technical barriers, such as CellProfiler [8,9], still require several hours of configuration depending on user expertise. These observations underscore the importance of developing robust, automated pipelines that reduce configuration time while maintaining segmentation accuracy. The approach presented in this study contributes to this goal by combining deep learning-based segmentation with an automated feature extraction workflow designed to minimize manual intervention.

A key aspect of morphological profiling is the reliability of downstream feature extraction. Since CellProfiler [8,9] remains the gold-standard implementation for Cell Painting [1] features, the consistency of the implemented feature extractors was evaluated relative to CellProfiler [8,9] outputs using the JUMP-CP [7] pilot dataset [24]. To ensure a fair comparison, CellProfiler [8,9] segmentation masks were used as input to both pipelines, and feature-level comparison was assessed using normalized mean squared error (MSE) and mean absolute error (MAE) metrics. The results demonstrate strong correlation across most feature groups, with particularly low deviations observed for shape- and texture-related features. Minor differences were observed for location- and neighbor-based features, primarily due to deviations in object handling near image borders and rounding implementation (Table 6).

Overall, low normalized MSE and MAE values indicate high consistency between the implemented feature extraction pipeline and CellProfiler [9] reference outputs. These findings validate the correctness of the feature extraction approach and suggest that the proposed workflow can reproduce existing methods. Importantly, the results demonstrate that deep learning-driven segmentation can be integrated within feature extraction pipelines while maintaining compatibility with widely adopted morphological profiling standards.

While the dataset captures substantial phenotypic variability, extending the workflow to structurally distinct cell types may require targeted adaptation. The workflow can be adapted to new datasets through retraining or fine-tuning, supporting its use across diverse biological contexts. The results highlight the potential of automated segmentation and feature extraction pipelines to reduce manual configuration overhead while preserving measurement accuracy. By minimizing dependence on handcrafted pipelines and parameter tuning, such approaches may improve reproducibility and scalability in high-throughput imaging workflows. This is particularly relevant for translational applications, where robust and standardized analysis pipelines are critical for leveraging morphological profiling.

## 5. Conclusions

This study presents an automated approach for Cell Painting [1] image analysis that enables feature extraction directly from raw microscopy data. Conventional image analysis pipelines often face challenges related to variability across imaging devices and experimental conditions, frequently requiring manual configuration. The presented computer vision-based workflow aims to improve robustness across heterogeneous datasets and reduce the need for extensive user intervention.

The proposed pipeline integrates segmentation and feature extraction into a unified workflow, simplifying morphological profiling and supporting scalable analysis in high-throughput settings. By reducing manual tuning and increasing automation, the approach contributes to more reproducible and accessible morphological profiling workflows. Automated morphological profiling pipelines may help streamline high-content imaging analysis and support data-driven research in drug discovery and precision medicine.

## Figures and Tables

**Table 1:** Experimental parameters of the CPG0000 dataset [24].

Parameter	Value
Cell lines	A549, U2OS
Number of plates	51
Plate format	384 well (r = 16) × (c = 24)
Replicates	2, 4
Time points	24, 48, 96, 144 h
Treatments	Compound (306), CRISPR sgRNA (335), ORF (175)
Gene targets	175+
Features	5794

**Table 2:** ImageXpress Micro XLS channels and stains in the Cell Painting assay [1].

Dye	Organelle or Cellular Component
Hoechst 33342	Nucleus (DNA)
Concanavalin A/Alexa Fluor 488 conjugate	Endoplasmic reticulum (ER)
SYTO 14	Nucleoli, cytoplasmic RNA (RNA)
Phalloidin/Alexa Fluor 568 + WGA/Alexa Fluor 555	F-actin cytoskeleton, Golgi, plasma membrane (AGP)
MitoTracker Deep Red	Mitochondria

**Table 3:** Distribution of features across measurement groups [26].

Feature Group	Number of Features
AreaShape	144
Correlation	300
Granularity	208
Intensity	225
Location	66
Neighbors	21
RadialDistribution	180
Texture	630

**Table 4:** Final segmentation results for eight-channel input (CEL-39).

Performance Metric	Nucleus Segmentation	Cell Segmentation
Labeled annotations	58,290	62,560
IoU	0.91	0.86
Precision (%)	98.27	96.60
Recall (%)	93.69	88.81
AP	0.92	0.86
False positives	961	2154

**Table 5:** Comparing the configuration time across different approaches [33].

Model Type	User Type	Configuration Time (h)
U-net models	Data scientist	20 h
CellProfiler novice	Novice image analyst	5 h
CellProfiler expert	Expert image analyst	3 h
Top-performing model	-	No configuration time needed

**Table 6:** Quality control results for feature extraction compared with CellProfiler [9] reference outputs.

Feature Group	Number of Features	MSE	MAE
AreaShape	55	$2.02\times 10^{-35}$	$1.73\times 10^{-19}$
Correlation	194	0.0043	0.0088
Granularity	128	$2.60\times 10^{-12}$	$2.77\times 10^{-7}$
Intensity	120	0.0134	0.0425
Location	32	0.0696	0.1577
Neighbor	15	0.0812	0.1705
RadialDistribution	96	0.0028	0.0377
Overall	640	0.0113	0.0298

## Data Availability

This study used data from the publicly available Cell Painting Gallery [21]. Derived datasets and intermediate results were generated in collaboration with KOLAIDO GmbH [28] and are not publicly available due to proprietary restrictions. The developed workflow is available as a Cell Painting assay on the IKOSA platform [28].
